# Refractory Penile Condyloma Acuminatum in an Elderly Man Receiving Vedolizumab and Azathioprine

**DOI:** 10.7759/cureus.97712

**Published:** 2025-11-24

**Authors:** Sohei Iwagami, Haruka Miyai, Masaya Nishihata

**Affiliations:** 1 Urology, Wakayama Medical University Hospital, Wakayama, JPN; 2 Urology, Kishiwada Tokushukai Hospital, Kishiwada, JPN

**Keywords:** azathioprine, condyloma acuminatum, infection, reactivation of hpv, vedolizumab

## Abstract

We report a case of an 83-year-old man who developed treatment-resistant penile condyloma acuminatum while receiving vedolizumab and azathioprine for ulcerative colitis. The patient had not engaged in sexual intercourse for more than five years. Laboratory findings revealed mild immunosuppression with decreased B-cell lymphocytes and low immunoglobulin A levels. The combined use of vedolizumab and azathioprine may have impaired genital mucosal immunity, leading to reactivation of latent human papillomavirus (HPV) infection. To our knowledge, condyloma acuminatum developing during vedolizumab therapy has rarely been reported, and this case suggests a potential association between vedolizumab and HPV reactivation.

## Introduction

Condyloma acuminatum has an annual incidence of 160-289 cases per 100,000 population. It is caused by human papillomavirus (HPV) infection and presents as genital, perianal, or oral verrucae characterized by epithelial proliferation [1].

Most patients are young, although rare cases occur in older adults. Disease onset may result not only from sexual transmission but also from reactivation of latent HPV infection due to advanced age or immunosuppression, such as that associated with human immunodeficiency virus (HIV) or immunosuppressive therapy [2,3].

There are almost no reports on the effects of vedolizumab, an agent used to treat ulcerative colitis, on extraintestinal mucosal immunity. This case represents the first suspected clinical report of latent HPV reactivation during combined azathioprine and vedolizumab therapy.

## Case presentation

**Figure 1:**
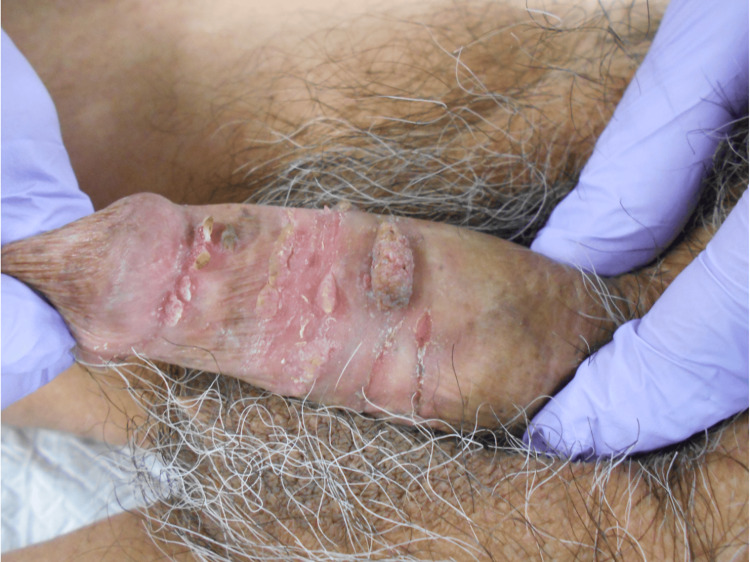
Physical findings Cauliflower-like genital warts formed on the mucosa from the coronal sulcus of the penis to the inner layer of the prepuce.

An 83-year-old male presented with genital warts persisting for one month and was clinically diagnosed with condyloma acuminata. He had received imiquimod for four weeks without improvement, and the lesions continued to enlarge, leading to referral to our department. The patient had a five-year history of ulcerative colitis treated with azathioprine. Owing to disease recurrence, vedolizumab was added two years earlier, and no further complications with ulcerative colitis were noted. His last reported sexual intercourse occurred more than 20 years ago, when he was treated for gonorrhea. On further review, including oral and anal sex, he disclosed a history of oral sex five to six years earlier. Visual examination revealed cauliflower-like verrucae on the glans and dorsal penis (Figure 1).

**Figure 2:**
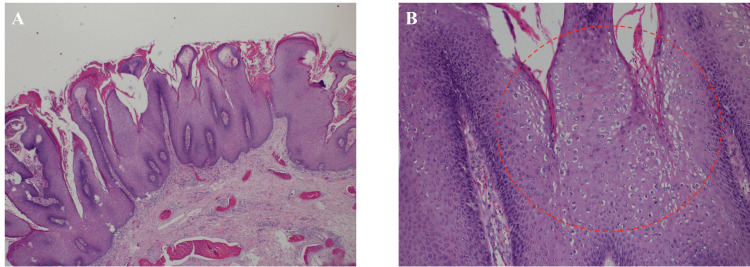
Pathological findings (A) Papillary elevation of squamous epithelium (low power field). (B) Inside the papillary ridge of the lesion (red circle), koilocytosis with vacuolization around the cell nucleus can be seen (high-magnification view).

Screening for sexually transmitted disease (STD) tests, except condyloma acuminatum, including HIV and syphilis, was negative. Laboratory tests showed a leukocyte count of 5,000/μL (within normal limits) and a lymphocyte count of 980/μL (mildly reduced). Lymphocyte subset analysis demonstrated T cells at 74.8% (normal range: 58-84%) and B cells at 2.8% (normal range: 5-24%), indicating a relatively low B cell percentage. The CD4/8 ratio was normal at 1.4 (normal range: 0.6-2.6). Immunoglobulin levels were IgG of 1,029 mg/dL, immunoglobulin A (IgA) of 87 mg/dL, and IgM of 98 mg/dL, with IgA slightly below the normal range (90-400 mg/dL). The albumin level was slightly low, but no other remarkable conditions were observed (Table 1). The patient underwent surgical excision with electro-incineration. Histopathological evaluation revealed papillary epithelial lesions with koilocytosis, consistent with condyloma acuminatum (Figure 2).

**Table 1:** Laboratory tests Immunoglobulins: IgG, IgA, and IgM

Laboratory tests	Test values (normal range)
		Leukocyte counts, 10^3^/μL	5.0 (3.3-8.6)
Hemoglobin, g/dL	14.5 (13.7-16.8)
Platelet, 10^4^/μL	17.2 (15.8-34.8)
Lymphocyte count, /μL	980 (1000-4800)
Neutrophils, /μL	2805 (1500-6000)
Monocytes, /μL	425 (200-950)
Eosinophils, /μL	400 (70-450)
Basophils, /μL	40 (0-300)
Total Bilirubin, mg/dL	0.6 (0.4-1.5)
Aspartate aminotransferase, U/L	27 (13-30)
Alanine aminotransferase, U/L	17 (10-42)
Albumin, g/dL	3.9 (4.1-5.1)
Serum creatinine, mg/dL	0.9 (0.6-1.0)
C-reactive protein, mg/dL	0.06 (0-0.14)
T cells	74.8 (58-84)
B cells	2.8 (5-24)
CD4	51.6 (24.3-49.7)
CD8	35.9 (18.4-49.0)
CD4/8, ratio	1.4 (0.6-2.6)
Immunoglobulin, mg/dL	
IgG	1029 (820-1740)
IgA	87 (90-400)
IgM	98 (31-200)

Recurrence occurred within three weeks, necessitating repeat surgical excision. The patient is scheduled for outpatient follow-up for at least one year.

## Discussion

The interval between HPV infection and wart formation in condyloma acuminatum ranges from four weeks to 18 months [4], although approximately 90% of cases remain subclinical without visible lesions [5]. Among HPV-infected patients, 70% clear infection within one year and 90% within two years, while the remainder develop latent infection [6]. Reactivation of latent HPV has been reported in association with immunosuppression due to HIV infection, advanced age, or immunosuppressive therapy [2,3].

Azathioprine is known to reduce T cells, B cells, and natural killer cells while suppressing immunoglobulin production, including IgA, which is critical for mucosal immunity [7-9]. In this case, the patient demonstrated mild lymphopenia, a marked reduction in B cells, and low IgA levels, indicating impaired humoral and mucosal immune protection against HPV. It is thought that reduced memory B cells may have impaired the rapid antibody response in the penile mucosa, allowing reactivation of latent HPV and the development of warts on the coronal sulcus and inner prepuce [10]. Reports of HPV reactivation during azathioprine monotherapy are limited; however, the recurrence of warts after initiation of vedolizumab in this case suggests that vedolizumab may have also contributed.

Vedolizumab, an α4β7 integrin antagonist, blocks adhesion to mucosal addressin cell adhesion molecule-1 (MAdCAM-1), thereby inhibiting lymphocyte trafficking to the gastrointestinal tract [11,12]. Although generally considered gut-selective, extraintestinal effects such as varicella-zoster virus reactivation and Kaposi’s sarcoma have been reported [13-15]. Experimental studies show that MAdCAM-1 may be expressed in other mucosal tissues, including genital-associated lymphoid sites, particularly during inflammation [16-18]. We hypothesize that vedolizumab, in combination with azathioprine, may have contributed to impaired genital mucosal immunity, leading to reactivation of latent HPV in this patient. The genital mucosa of older adults is often chronically inflamed [19], and MAdCAM-1 expression in such genital mucosa and regional lymph nodes may inhibit lymphocyte migration, further promoting HPV reactivation.

This study has limitations. First, while the possibility of HPV reactivation due to vedolizumab is suspected, it remains purely speculative, and the specific mechanism has not been elucidated. Second, it is difficult to prove whether patients actually engaged in sexual intercourse or similar activities. We endeavored to obtain information from patients through repeated interviews and the establishment of trust. Furthermore, regarding the mechanism, similar reports are scarce, and new reports and further research are anticipated in the future.

To our knowledge, condyloma acuminatum developing during vedolizumab therapy has not previously been documented. In this case, azathioprine-induced B-cell and IgA reduction, together with vedolizumab-associated suppression of T-cell trafficking to the genital mucosa, likely disrupted local immunity and enabled HPV reactivation. Although causality cannot be established, these findings suggest a potential new adverse effect of vedolizumab acts selectively on the intestinal tract and azathioprine combination therapy. Patients receiving vedolizumab should be monitored closely for HPV reactivation, which may increase the risk of treatment resistance and refractory disease, and consideration should be given to changing or discontinuing vedolizumab. This case also emphasizes the importance of considering sexually transmitted infections, including HPV, in older adults with remote or limited sexual histories, and highlights the need for thorough sexual history-taking in immunocompromised patients.

## Conclusions

This case illustrates that vedolizumab and azathioprine therapy may impair mucosal immunity and facilitate the reactivation of latent HPV infection, leading to treatment-resistant penile condyloma acuminatum. Although causality cannot be proven, clinicians should be aware of this possible association in immunosuppressed elderly patients. Careful monitoring for viral infections is warranted during vedolizumab therapy.

## References

[1] Patel H, Wagner M, Singhal P, Kothari S (2013). Systematic review of the incidence and prevalence of genital warts. BMC Infect Dis.

[2] Sindhuja T, Bhari N, Gupta S (2022). Asian guidelines for condyloma acuminatum. J Infect Chemother.

[3] Maglennon GA, McIntosh PB, Doorbar J (2014). Immunosuppression facilitates the reactivation of latent papillomavirus infections. J Virol.

[4] Doorbar J (2023). The human Papillomavirus twilight zone - latency, immune control and subclinical infection. Tumour Virus Res.

[5] Sendagorta-Cudós E, Burgos-Cibrián J, Rodríguez-Iglesias M (2019). Genital infections due to the human papillomavirus. Enferm Infecc Microbiol Clin (Engl Ed).

[6] Veldhuijzen NJ, Snijders PJ, Reiss P (2010). Factors affecting transmission of mucosal human papillomavirus. Lancet Infect Dis.

[7] Tiede I, Fritz G, Strand S (2003). CD28-dependent Rac1 activation is the molecular target of azathioprine in primary human CD4+ T lymphocytes. J Clin Invest.

[8] Lord JD, Shows DM (2017). Thiopurine use associated with reduced B and natural killer cells in inflammatory bowel disease. World J Gastroenterol.

[9] Levy J, Barnett EV, MacDonald NS, Klinenberg JR, Pearson CM (1972). The effect of azathioprine on gammaglobulin synthesis in man. J Clin Invest.

[10] Dhanushkodi NR, Prakash S, Srivastava R (2022). Antiviral CD19+CD27+ memory B cells are associated with protection from recurrent asymptomatic ocular herpesvirus infection. J Virol.

[11] Feagan BG, Rutgeerts P, Sands BE (2013). Vedolizumab as induction and maintenance therapy for ulcerative colitis. N Engl J Med.

[12] Loftus EV Jr, Feagan BG, Panaccione R (2020). Long-term safety of vedolizumab for inflammatory bowel disease. Aliment Pharmacol Ther.

[13] Karlqvist S, Sachs MC, Eriksson C (2024). Comparative risk of serious infection with vedolizumab vs anti-tumor necrosis factor in inflammatory bowel disease: results from nationwide Swedish registers. Am J Gastroenterol.

[14] Celada-Sendino M, Carballo-Folgoso L, de Francisco R, Pérez-Martínez I, Castaño-García A, Riestra S (2023). Varicella zoster virus encephalitis: a potentially serious complication during treatment with vedolizumab. Rev Esp Enferm Dig.

[15] Ajao SO, Jayasingam R, Shaaban H (2021). Iatrogenic Kaposi's sarcoma unmasked by vedolizumab in a patient with ulcerative colitis and well-controlled human immunodeficiency virus: a case report. Int J Crit Illn Inj Sci.

[16] Blaisdell A, Zhou Y, Kattah MG, Fisher SJ, Mahadevan U (2022). Vedolizumab antagonizes MAdCAM-1-dependent human placental cytotrophoblast adhesion and invasion in vitro. Inflamm Bowel Dis.

[17] Ando T, Langley RR, Wang Y, Jordan PA, Minagar A, Alexander JS, Jennings MH (2007). Inflammatory cytokines induce MAdCAM-1 in murine hepatic endothelial cells and mediate alpha-4 beta-7 integrin dependent lymphocyte endothelial adhesion in vitro. BMC Physiol.

[18] Soderberg KA, Linehan MM, Ruddle NH, Iwasaki A (2004). MAdCAM-1 expressing sacral lymph node in the lymphotoxin beta-deficient mouse provides a site for immune generation following vaginal herpes simplex virus-2 infection. J Immunol.

[19] Dayal S, Sahu P (2016). Zoon balanitis: a comprehensive review. Indian J Sex Transm Dis AIDS.

